# Politicizing mask-wearing: predicting the success of behavioral interventions among republicans and democrats in the U.S.

**DOI:** 10.1038/s41598-022-10524-1

**Published:** 2022-05-09

**Authors:** Eugen Dimant, Elena Giulia Clemente, Dylan Pieper, Anna Dreber, Michele Gelfand, Behavioral Science Units Consortium, Michael Hallsworth, Aline Holzwarth, Piyush Tantia

**Affiliations:** 1 Center for Social Norms and Behavioral Dynamics, University of Pennsylvania, Philadelphia, USA; 2 CESifo, Munich, Germany; 3 Stockholm School of Economics, Stockholm, Sweden; 4 University of Maryland, College Park, USA; 5 University of Innsbruck, Innsbruck, Austria; 6 Stanford University, Stanford, USA; 7 Behavioral Insights Team, New York, NY USA; 8 Center for Advanced Hindsight, Durham, NC USA; 9 ideas42, New York, NY USA

**Keywords:** Psychology, Human behaviour

## Abstract

Scientists and policymakers seek to choose effective interventions that promote preventative health measures. We evaluated whether academics, behavioral science practitioners, and laypeople (*N* = 1034) were able to forecast the effectiveness of seven different messages compared to a baseline message for Republicans and Democrats separately. These messages were designed to nudge mask-wearing attitudes, intentions, and behaviors. When examining predictions across political parties, forecasters predicted larger effects than those observed for Democrats compared to Republicans and made more accurate predictions for Republicans compared to Democrats. These results are partly driven by a lack of nudge effects on Democrats, as reported in Gelfand et al. (J Exp Soc Psychol, 2021). Academics and practitioners made more accurate predictions compared to laypeople. Although forecasters' predictions were correlated with the nudge interventions, all groups overestimated the observed results. We discuss potential reasons for why the forecasts did not perform better and how more accurate forecasts of behavioral intervention outcomes could potentially provide insight that can help save resources and increase the efficacy of interventions.

## Introduction

The COVID-19 outbreak has had an unprecedented impact on our lives. Until vaccines became available, the combination of social distancing and wearing masks was the most promising way to reduce the spread of COVID-19 according to the CDC^1^. Behavioral science has taken a center stage in encouraging adherence to these guidelines and reducing vaccination hesitancy^2–7^.

Since the beginning of this pandemic, polls in the U.S. have indicated a growing political divide over mask-wearing attitudes, which has been exacerbated by the existing stark political polarization^6,8–15^. Because such preventive health measures are more effective the more people follow them, the existing partisan divide creates challenges from a policy perspective. An additional layer of complexity is added because Republicans and Democrats can be expected to respond to health-related messaging and policy interventions in different ways^4,11,16–18^. But to what extent can these differences actually be predicted? And is one’s predictive ability shaped by one's own political ideology and behavioral science savviness?

In this paper, we evaluate the extent to which academics, practitioners, and laypeople are able to correctly forecast the effectiveness of behavioral interventions aimed at promoting mask-wearing. We look at forecasts of this tournament along four dimensions for which we have outcome data: two behavioral measures (signing a pledge to commit to wearing a mask and willingness to share the pledge on social media) and two attitudinal measures (mask wearing attitudes and mask wearing intentions). Recent literature stresses the scientific benefits of collecting predictions of scientific outcomes^9,19–24^. In these studies, participants—mainly researchers—are asked to predict replication outcomes^19,22^ or new outcomes^21^ with either prediction markets, surveys, or structured elicitation protocols^25^. This research shows that forecasts are surprisingly accurate in predicting which research replicates successfully and which interventions succeed (cf. see the Many Labs 2 studies for instances of unsuccessful predictions for effect size)^26^. We extend this literature and utilize forecasts to generate collective estimates of which nudges are successful and which are not in a previously unexplored domain (mask-wearing during the COVID-19 pandemic) for both Democrats and Republicans separately.

## Nudges to wear a mask

We collected forecasts on the outcomes of mask-wearing behavioral interventions for Republicans and Democrats based on the nudge study of a companion registered report^11^. Building on both moral foundation theory^27,28^ that examines moral framing as a means to reduce attitudinal polarization between political groups^17^, along with wise intervention theory^29^ that targets the specific psychological mechanism(s) that may underlie why Republicans or Democrats are reluctant to wear facial masks or coverings, the goal of the intervention study was to evaluate the effectiveness of 7 nudge interventions relative to a baseline in a representative U.S. sample of Republicans and Democrats (*N* = 4931)^11^. First, based on moral foundations theory^27,28^ we developed four different moral-frame interventions, including individual harm, group harm, ingroup loyalty, and purity^27,28^. We specifically examined whether messages that reflected moral foundations such as ingroup-loyalty and purity would be more effective for Republicans, while those that focus on harm would be more effective for Democrats. Drawing on research on moral motives^30^, we also included a hybrid group harm condition that focused on both concern for the group and concern for personal harm, which could be effective among both Republicans and Democrats. Second, we included 3 additional nudges specific to the COVID-19 pandemic that argued that it is important to wear masks because the virus is a serious threat, because scientific evidence suggests they are effective, or because doing so will help revive the economy, all of which showed Republican-Democrat political divisions during COVID-19.

The nudges were designed and pilot tested to promote four specific outcomes: mask-wearing attitudes, intentions to wear a mask, pledging to wear a mask, and copying the link to the pledge website to share on social media. The latter two outcomes were considered proxies for behavior. This study had high statistical power (95%) to detect small effects equivalent to *f*^2^ = 0.003 (for a MANOVA on attitudes and intentions) or *OR* = 1.20 (for a χ^2^ test on signing and sharing the pledge) including the interaction between the nudge conditions and political party (α = 0.01).

In our companion paper, we found strong partisan effects such that Republicans had more negative attitudes toward masks, lower intentions to wear masks, and were less likely to sign and share a pledge to wear a mask compared to Democrats^11^. However, there was no evidence that 7 nudge interventions were effective at promoting mask-wearing among Democrats or Republicans in the U.S. relative to a baseline condition (omnibus test *p*s > 0.01). These results suggested that promoting mask-wearing attitudes and behavior is challenging. The nudges also remained ineffective when controlling for demographic variables and when using an alternative baseline condition. In this study, we instead focused on whether these outcomes can be predicted using three groups of forecasters: laypeople, academics, and practitioners.

## The present study: forecasting nudges

While the forecasting ability of laypeople, academics, and practitioners have previously been evaluated separately^19,31,32^, our study is one of the first to systematically evaluate them simultaneously within the same forecasting paradigm (see also)^33^. Capturing potential differences in forecasting abilities is valuable for multiple reasons. For example, researchers and policymakers constantly make decisions regarding which intervention to implement. Beliefs that are at odds with the actual success of a nudge could prove costly from a welfare perspective if the wrong intervention is selected. While accurate empirically informed beliefs and predictions increase the chances of implementing effective interventions, overestimating the effectiveness of the selected interventions runs the risk of underpowering empirical studies and overvaluing the strength of the results. In contrast, underestimating the selected interventions runs the risk of ignoring potentially effective interventions.

## Hypotheses

We set forth a number of testable hypotheses to guide our empirical strategy. Our evaluation centers around examining forecasters’ ability to predict mask-wearing nudges along multiple dimensions, including: the type of nudge tested, the type of outcome measured, the political affiliation of the nudgee, and the behavioral science background of the forecaster. We pre-registered our hypotheses on the Open Science Framework (https://osf.io/zwpbr/). Hypothesis 1 is a directional hypothesis based on previous literature suggesting that forecasted predictions are positively associated with the observed results^19–24,34^. Hypotheses 2–6 are non-directional and reflect that we assumed that there was variation across groups and outcomes, but we remained neutral towards the direction of the forecasters’ predictions:

### Hypothesis 1

There is a positive association between the forecasters’ predicted effects and the observed results for nudge predictions (*H1a*) and for the predictions on the effect of the baseline condition (*H1b*).

### Hypothesis 2

The predicted nudge effect sizes for Democrats differ from the predicted nudge effects for Republicans.

### Hypothesis 3

The accuracy of the predictions differs for Democrats and Republicans across the nudge interventions, where accuracy is defined as the squared prediction error.

### Hypothesis 4

Forecasters’ under/over-estimate the observed nudge effects in the pooled sample of predictions (*H4a*) as well as separately for the predictions of Democrat (*H4b*) and Republican (*H4c*) samples.

### Hypothesis 5

The accuracy of predictions differs for predictions of nudge effects for attitudes, intentions, and the two behavioral tasks.

### Hypothesis 6

The accuracy of predictions of nudge effects differs between the three categories of participants—laypeople, academics, and practitioners.

The phrasing of some hypotheses may differ from the pre-registration document to improve their clarity and conciseness; however, we did not alter their meaning or make any substantive changes. Supplemental Information part E describe deviations from the Pre-Registration plan.

## Methods

Participants’ forecasts of the nudge conditions were incentivized through a raffle system. Participants received one ticket for each prediction if their guess was within + / − 0.10 points of the observed effect size. For the prize, we distributed ten $50 Amazon gift cards by randomly selecting from the pool of raffle tickets, with 10 participants being paid.

We conducted our study in the spring of 2021 and collected data from three sources to address our research questions: purchasing access to the sample that the company Qualtrics maintains, our own academic networks, and the professional networks of our behavioral insights partners. To obtain a diverse sample of laypeople, we collected responses using the diverse panel of survey takers that the company Qualtrics maintains. We signed a contract with Qualtrics that allowed us to tap into their pool of participants that was representative of the U.S. population. This representative laypeople sample (*n* = 611) consisted of 294 Republicans and 317 Democrats and fulfilled quotas on region, gender, age, race, and education level matched to the demographics of the specific political group in the U.S. population. To confirm that participants identified as either Republican or Democrat, respondents were asked to indicate their party affiliation in the beginning of the survey by choosing among the two. We added additional laypeople to the sample from our professional networks (*n* = 103).

Using the professional social networks of the authors, we also collected data from academics and behavioral science practitioners. Data for academics (*n* = 199) and practitioners (*n* = 121) was collected through academic mailing lists (e.g., ESA and JDM), Twitter, Social Science Prediction Platform (https://socialscienceprediction.org/), and our consortium coauthors at the respective units who gave us access to their employees (i.e., Behavior Change for Good Initiative, Behavioral Insights Team, ideas42, Center for Advanced Hindsight, and Office of Evaluation Sciences). Forecasters recruited through professional social networks were also asked to provide their political affiliation (as either “Republican”, “Democrat”, “Independent” or “Other”) while completing the survey.

To categorize academics and practitioners, we asked which option best describes participants’ current professional identity (see Supplemental Information part B for the exact measure and categorization strategy that we used). In sum, we were able to collect data from a total of 1034 participants. Table 1 presents sample characteristics for the Qualtrics and the professional networks sample. See Supplemental Information part C for sample characteristics and demographics for academics and practitioners separately.

**Table 1:** Sample demographics, qualtrics versus professional networks.

Name	Qualtrics (n = 611)	Professional networks (n = 423)
Age in years	53.97 (17.44)	33.90 (10.38)
Family income*	2.41 (1.33)	3.71 (1.60)
Socioeconomic status (SES)	5.99 (2.24)	8.26 (1.69)
Ideology (liberal to conservative)	5.51 (2.64)	3.09 (1.45)**
**Gender**		
Woman	341 (56%)	185 (44%)
Man	270 (44%)	235 (56%)
Other	–	3 (1%)
**Race**		
White	436 (71%)	320 (76%)
Black	75 (12%)	4 (1%)
Hispanic	60 (10%)	22 (5%)
Asian	32 (5%)	51 (12%)
Multiracial	5 (1%)	12 (3%)
Pacific Islander	–	1 (< 1%)
Other	3 (< 1%)	13 (3%)
**Highest education**		
High school	174 (28%)	3 (1%)
College (no degree)	159 (26%)	10 (2%)
Graduate (4-year)	131 (21%)	104 (25%)
Professional degree (Ph.D., M.A., etc.)	93 (15%)	301 (72%)
Technical degree	51 (8%)	–
Grammar school	1 (< 1%)	1 (< 1%)
Other	–	1 (< 1%)
**Area**		
Urban	183 (30%)	260 (62%)
Suburban	285 (47%)	131 (31%)
Rural	143 (23%)	32 (8%)
**Self-identification**		
Layperson	484 (79%)	103 (24%)
Academic	19 (3%)	199 (47%)
Practitioner	108 (18%)	121 (29%)

Values are means (SDs) or counts (frequencies) unless otherwise noted.

*1 = “Below 30k”, 2 = “30–60k”, 3 = “60–90k”, 4 = “90–120k”, 5 = “Above 120k”. **Missing two participants’ responses.

Participants in this study were presented with all of the original 7 nudge interventions in a random order. The implemented nudges—all of which were motivated by existing literature—ranged from moral-frame interventions (including individual harm, group harm, loyalty, and purity) to framing interventions that tapped into other psychological mechanisms (including the economic impact of not wearing masks, the threat of COVID-19 indicated by mortality statistics, and scientific evidence in favor of masks). All intervention messages are summarized in Table 2 (more detailed content is included in the Supplementary Material). Participants were asked to predict the effectiveness of the nudge interventions in the original nudge study^11^ relative to a no-intervention baseline on the frequency of holding positive attitudes about masks, intending to wear a mask, signing a pledge to wear a mask, and sharing a pledge to wear a mask on social media. Participants were asked to predict whether a nudge would make “no difference” (d = 0) or result in positive views being held “more often” (max *d* = 1.0) or “less often” (min *d* = 1.0). Each forecaster was asked to provide 64 predictions that were subdivided into two groups: 8 predictions focusing on the effect of the baseline condition on attitudes, intentions, and behaviors and 56 predictions focusing on effects of the 7 nudge messages relative to the baseline condition, which were split between forecasting their effectiveness on Democrats versus Republicans. These predictions are the main focus of our forecasting study. See the Supplemental Information Part D to view the exact formatting of the forecasted predictions in our survey.

**Table 2:**
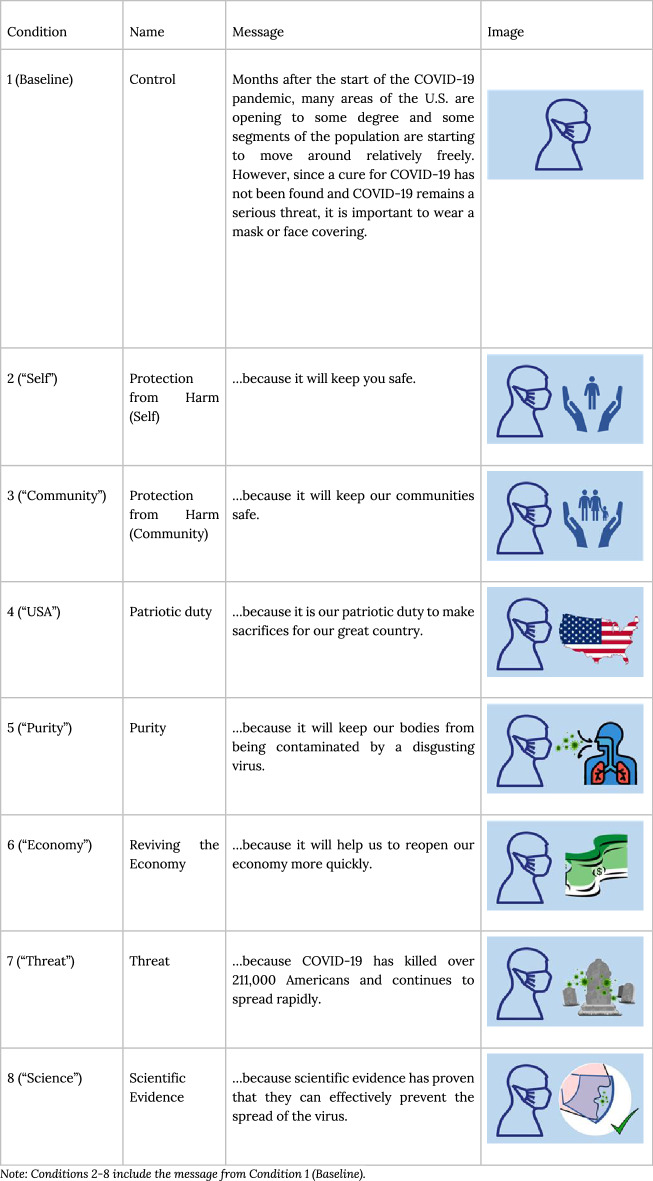
Original nudge conditions Gelfand et al.^11^.

Since the original intervention study focused on the differential effectiveness of the tested nudges on Democrats and Republicans, we accounted for one’s political party in our study. In addition, we explored whether a forecaster’s own political affiliation correlates with the precision of their prediction. The main analysis pooled predictions from the three categories of forecasters, but we also tested whether these categories differ in their accuracy.

All methods were carried out in accordance with relevant guidelines and regulations at the University of Maryland. In addition, all experimental protocols were approved by the University of Maryland. Informed consent was obtained from all subjects. An IRB approving the study was obtained through the University of Maryland.

### Execution and approval

All methods were carried out in accordance with relevant guidelines and regulations at the University of Maryland. In addition, all experimental protocols were approved by the University of Maryland.

### Informed consent

Informed consent was obtained from all subjects and an IRB was obtained through the University of Maryland.

## Materials, data, and code

Our study pre-registration, survey materials, data, and analysis script code are publicly available on the Open Science Framework (https://osf.io/zwpbr/). We follow the pre-registered analysis plan unless stated otherwise (see Supplemental Information part E for details on how our analyses deviated from the pre-registered analysis plan).

## Results

Before testing our hypotheses, we calculated standardized effect sizes in Cohen’s *d* for the observed results found in the original nudge study^11^. Specifically, we calculated the effect sizes first by calculating the means, standard deviations, standard errors, and sample sizes for the intervention (vs. the baseline) conditions on each outcome across the entire sample and by political party. We then inputted these values into an effect size calculator for an independent *t*-test^35^. We also retrieved the mean responses for the outcomes in the baseline condition to compare with forecasters’ beliefs about the strength of the baseline condition. We then calculated squared prediction error by subtracting forecasters' predictions from the observed results and squaring the difference, and absolute prediction error by taking the absolute value of the difference, according to the formulas:

$${\text{SPE}}_{{{\text{is}}}} = (BELIEFS_{is} {-}EFFECTS_{s} ){^2}$$

$${\text{APE}}_{{{\text{is}}}} = |BELIEFS_{is} {-}EFFECTS_{s} |$$

where *SPE*_*is*_ is the squared prediction error and APE_is_ is the absolute prediction error, BELIEFS_is_ is the predicted effect size and EFFECTS_s_ is the observed effect size, for nudge *s* and forecaster *i*. Throughout the rest of the paper, ‘prediction error’ indicates squared prediction error, unless explicitly stated otherwise. See Supplemental information part F for the data used to calculate the observed effect sizes.

To test Hypothesis 1a, we ran the following individual-level OLS regression:

$$EFFECTS_{s} = \beta_{0} + \beta_{{1}} BELIEFS_{is} + FE_{i} + \varepsilon_{is}$$

where *EFFECTS*_*s*_ is a continuous variable of the observed effect size for each of the seven nudges s; *BELIEFS*_*is*_ is a continuous variable indicating the predicted effect size of forecaster *i* for nudge *s*; *FE*_*i*_ is the set of forecaster fixed effects, included to control for unobserved heterogeneity in predictions across individual forecasters. Standard errors are clustered at the forecaster level, to take into account the correlation among the different predictions made by each forecaster. The total number of observations is therefore the number of forecasters times the number of nudges, i.e. 1034 × 7 = 7238.

The results showed a positive, albeit small, statistically significant association between forecasters’ predictions and the observed results in the nudge predictions when including individual fixed effects, in line with our directional hypothesis (β = 0.03, *SE* = 0.005, *t*(7236) = 6.26, *p* < 0.001). The results indicate that there is a positive relationship between forecasters’ beliefs and observed results: higher predicted nudge effects are associated with higher observed nudge effects, although most of the original effects were not statistically significantly different from zero (pre-registered α = 0.01). However, in a robustness test, the Pearson correlation between the aggregate predictions and observed effect sizes for the 56 forecasted effects was not statistically significant (r(56) = −0.03, t(54) = −0.21, *p* = 0.83).

In the survey, forecasters were also asked to predict mask-wearing attitudes, intentions and behaviors of the participants in the original study presented with the no-intervention control message (Table 2, first row). Forecasters were partially able to predict the baseline attitudes, intentions, and the two pledging behaviors for the pooled sample of Republicans and Democrats in the original study. Hypothesis 1b was tested with the same regression as hypothesis 1a, but using baseline predictions only. In line with our directional hypothesis for H1b, there was a positive and statistically significant association between the predictions (beliefs) of forecasters and the observed results in the baseline predictions after controlling for individual fixed effects (β = 0.68 , *SE* = 0.009, *t*(4,134) = 77.34, *p* < 0.001). Forecasters’ predictions also explained a statistically significant proportion of variance in the observed results (*R*^2^ = 0.59, *R*^2^_*adj*_ = 0.46, (1; 1,033) = 5982.18, *p* < 0.001). Estimating the Pearson correlation between aggregate forecasted and observed results for baseline predictions supported this statistically significant positive association (r(8) = 0.82, t(6) = 3.63, *p* = 0.01).

**Figure 1:**
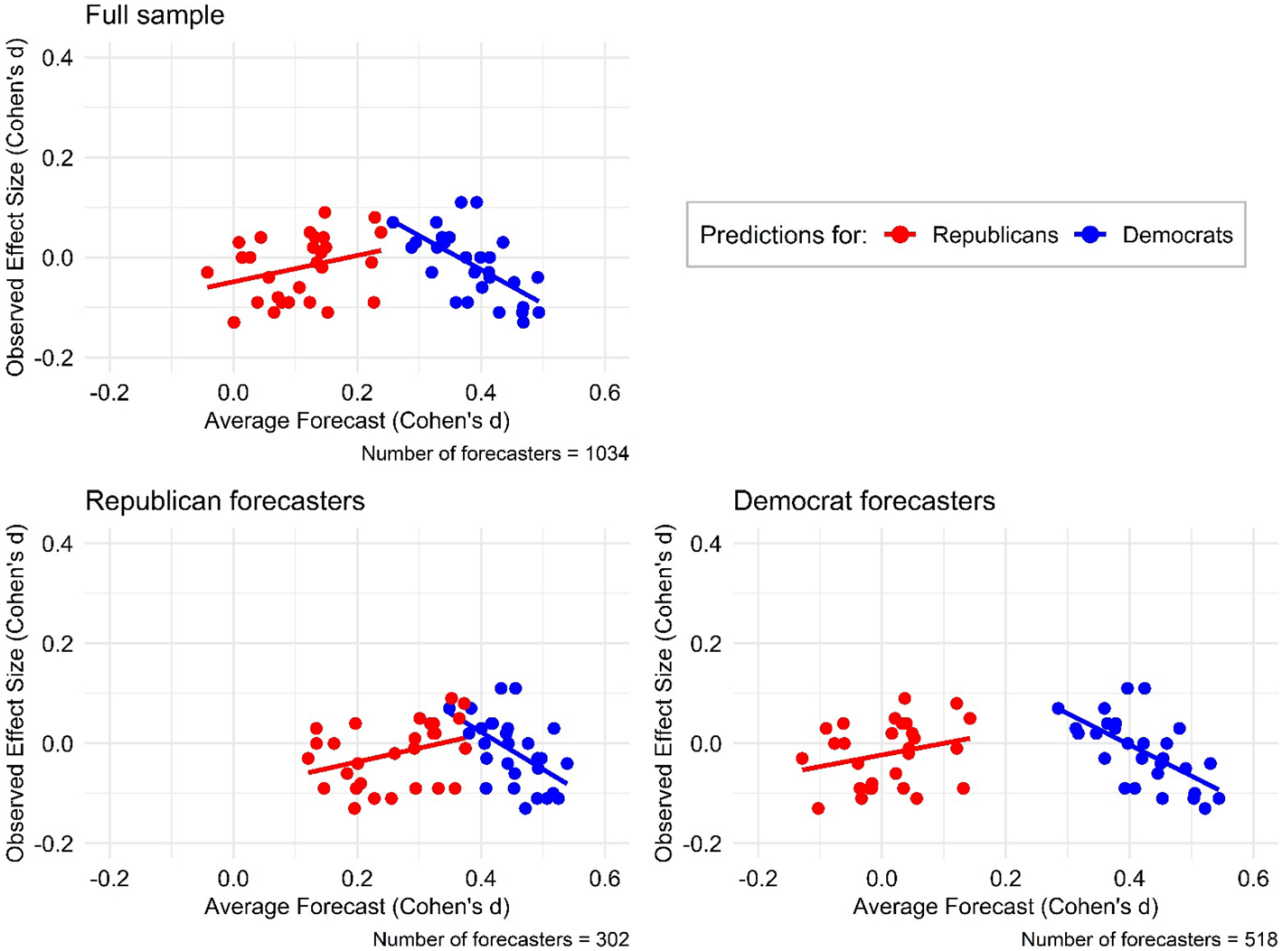
Correlations between forecasted and observed effect by political party of forecaster and nudgee. *Note*: Correlation between observed and predicted effect sizes (in terms of Cohen’s d) for the 56 forecasting questions in the forecasting survey, differentiated by color for forecasts of Republican (red) and Democrat (blue) nudgees. The top panel includes the full sample of forecasters. The bottom panel shows the correlation separately for Democrat and Republican forecasters.

Next, we examined if forecasters expected nudge interventions to affect Republicans and Democrats differently in the original sample (Hypothesis 2). A paired t-test of the average of the 28 predicted nudge effects for Democrats and Republicans made by each forecaster showed that the two predicted effects were statistically significantly different (*mean difference* = 0.28, *t*(1,033) = 17.98, *p* < 0.001). Forecasters predicted that the effect of nudge interventions relative to the baseline conditions will be larger for Democrat participants compared to Republican participants in the original study. Figure 1 plots predicted and observed effect sizes separately for Democrats and Republicans for the full sample of forecasters and, as a non-pre-registered test, for the two subsamples of forecasters who identify as either Republicans or Democrats. As an additional, non-pre-registered test, we found that the correlation between aggregate forecasted and observed results is negative and statistically significant for Democrat predictions (r(28) = −0.63, t(26) = −4.12, *p* = 0.0003) and positive, but not statistically significant, for Republican predictions (*r*(28) = 0.30, *t*(26) = 1.63, *p* = 0.12) in the full sample of forecasters.

**Figure 2:**
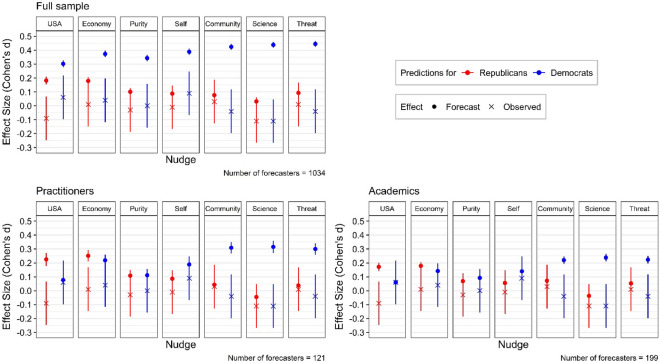
Forecasted and observed effects by nudge conditions and political party of nudgee. *Note*: Predicted (dot) and observed (cross) effect sizes (Cohen’s d) for the seven nudge predictions, differentiated by color for forecasts regarding Republican (red) and Democrat (blue) nudgees. The top panel includes the full sample of forecasters while the bottom panel shows effect sizes separately for academic and practitioner forecasters. Error bars represent the 95% confidence interval. “USA” indicates the “Patriotic duty” nudge; “Economy” indicates the “Reviving the Economy” nudge; “Self” indicates the “Protection from Harm (Self)” nudge; “Community” indicates the “Protection from Harm (Community)” nudge; “Science” indicates the “Scientific evidence” nudge.

For Hypothesis 3, we examined whether the accuracy of the predictions differs for the Democrat forecasts and Republican forecasts across all nudges. A paired *t*-test of the mean squared prediction error, as defined above, for Democrats and Republicans predictions for each forecaster indicated that the accuracy between the two sets of predictions were statistically significantly different (*mean difference* = 0.08, *t*(1,033) = 13.05, *p* < 0.001). Specifically, forecasters made more accurate predictions for Republicans compared to Democrats (i.e., the mean squared prediction error is higher for predictions for Democrats). This is not surprising, as forecasters expected larger effect sizes for Democrats relative to Republicans. Given that the observed effect sizes of the nudges in the original study were comparably small for both samples of participants, this resulted in higher prediction error for the forecasts of Democrats^11^. Figure 2 illustrates the average predicted (dot) and observed (cross) effect size for each of the seven nudges for predictions about Republicans (red) and Democrats (blue), for the full sample of forecasters and the two subsamples of practitioners and academics.

As a robustness check for Hypothesis 3, we used *absolute* prediction error as a measure of accuracy. We again found that the prediction error was statistically significantly different for forecasts for Democrats and Republicans and is higher for Democrats using a *t*-test (*mean difference* = 0.11, (1,033) = 16.78, *p* < 0.001).

For Hypothesis 4, we estimated the under/over-estimation of observed nudge effects. A *z*-test comparing the seven predicted nudge effect sizes for each forecaster to the mean observed nudge effects showed that forecasters overestimated the effect size in the pooled sample (*z* − *score* = 4.40, *p* < 0.001, H4a). Forecasters also overestimated the nudge effects for Democrats (*z* − *score* = 4.84, *p* < 0.001, H4b), while the difference between forecasted and observed nudge effects was not statistically significantly different from zero for forecasts for Republicans (*z* − *score* = 1.67, *p* = 0.09, H4c). In sum, forecasters overestimated the effects of the interventions for the full sample and Democrats but not for Republicans, relative to the baseline condition.

**Figure 3:**
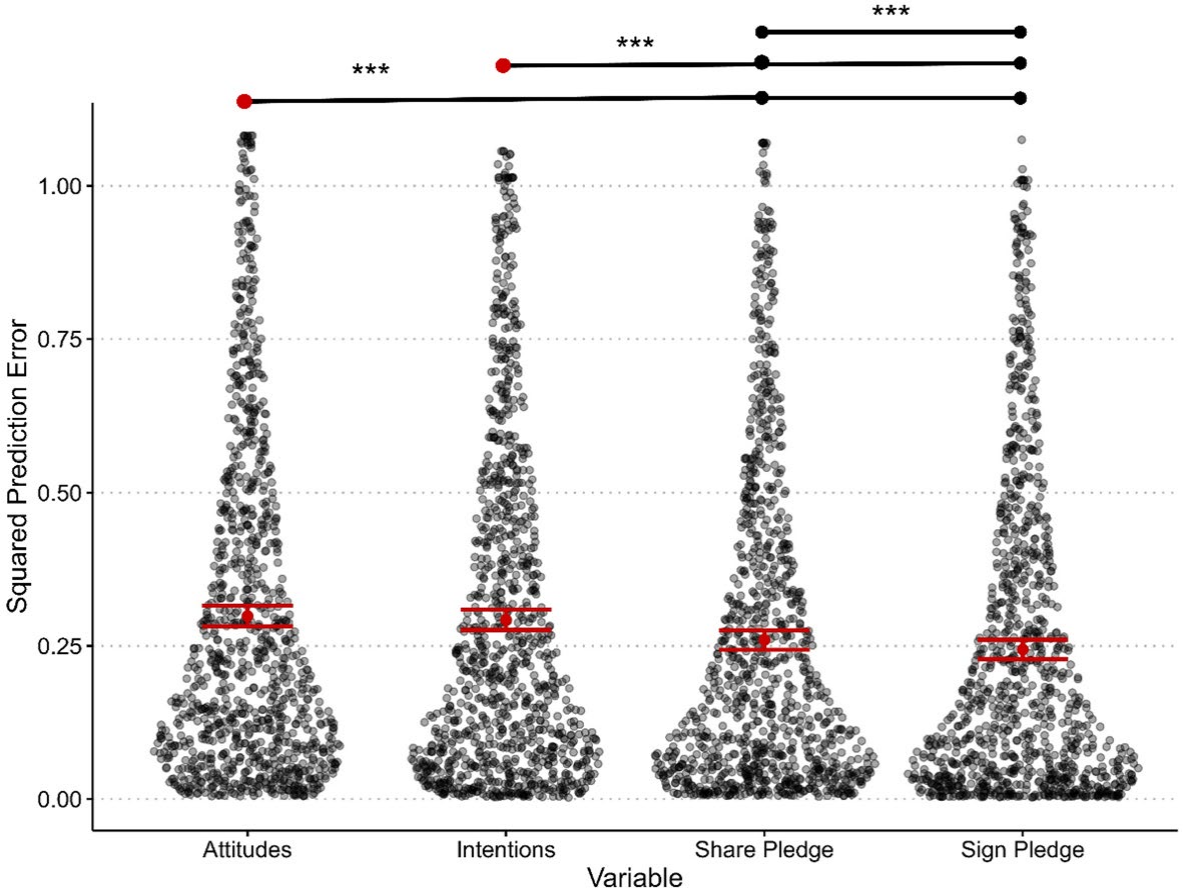
Prediction error across variables. *Note*: Variables ordered by largest to smallest prediction error (left to right). Black and transparent dots indicate the distribution of individual data points for prediction error across variables. Black dots above the distribution of data points represent variables that show statistically significant differences, whereas red dots above the distribution of data points represent reference variables for comparing statistically significant differences between more than two variables. Error bars represent the 95% confidence interval of the mean. *** p < .001.

For Hypothesis 5, we tested whether the accuracy of forecasters’ predictions (i.e., mean squared prediction error) differs for predictions of nudge effects on attitudes, intentions, and the two pledging behaviors. Figure 3 plots the prediction error across the four outcomes and the statistically significant pairwise comparisons. A repeated-measures ANOVA showed statistically significant within-subjects differences in prediction error between the four outcomes (f(1.83, 1,887.74) = 103.90, *p* < 0.001, ω^*2*^ = 0.007). Mauchly's test of sphericity indicated that the assumption of sphericity was violated (*p* < 0.001), which was corrected for using the Greenhouse–Geisser method. Post-hoc comparison *t*-tests, adjusting for a family of six estimates using the Holm-Bonferroni correction, revealed statistically significantly higher prediction error for attitudes compared to signing the pledge (*mean difference* = 0.06, *SE* = 0.004, *t*(1,033) = 15.03, *p*_ℎ*olm*_, < 0.001) and sharing the pledge (*mean difference* = 0.04, *SE* = 0.004, *t*(1,033) = 10.76, *p*_ℎ*olm*_ < 0.001). There was also statistically significantly higher prediction error for intentions compared to signing the pledge (*mean difference* = 0.05, *SE* = 0.004 , *t*(1,033) = 13.35, *p* < 0.001) and sharing the pledge (*mean difference* = 0.03, *SE* = 0.004, *t*(1,033) = 9.08, *p*_ℎ*olm*_ < 0.001). Furthermore, there was a statistically significantly higher prediction error for sharing the pledge compared to signing the pledge (*mean difference* = 0.02, *SE* = 0.004, t(1,033) = 4.27, *p*_ℎ*olm*_ < 0.001). There was, however, no statistically significant difference in prediction error between attitudes and intentions (*p*_ℎ*olm*_ = 0.09).

**Figure 4:**
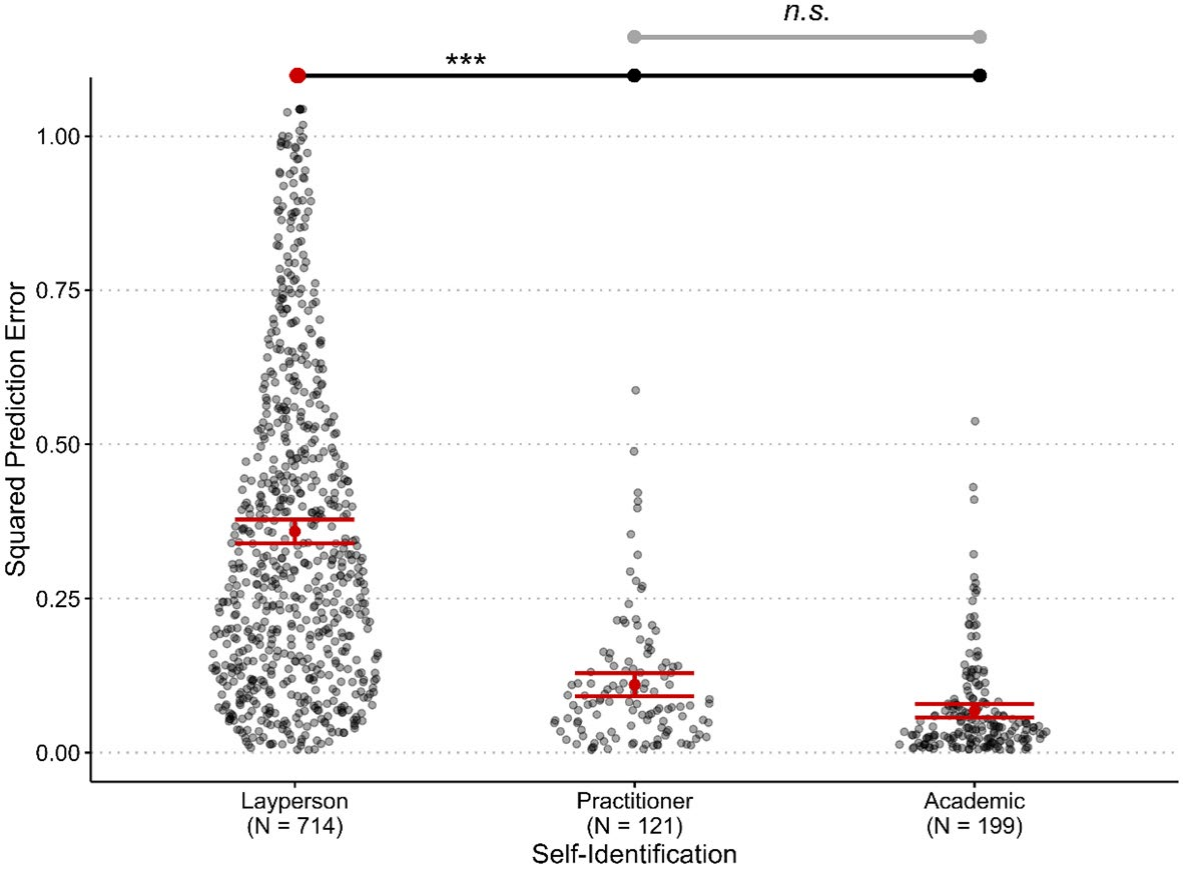
Prediction error by self-identified group. *Note*: Variables ordered by largest to smallest prediction error (left to right). Black and transparent dots indicate the distribution of individual data points for prediction error across categories. Black dots above the distribution of data points represent categories that show statistically significant differences, whereas red dots above the distribution of data points represent reference categories for comparing statistically significant differences between more than two categories. Gray dots and a gray line indicate a non-significant (n.s.) relationship. Error bars are the 95% confidence interval of the mean. ***p < .001.

For Hypothesis 6, we tested whether the accuracy of predictions of nudge effects differs between the three categories of participants—laypeople, academics, and practitioners. Figure 4 plots the prediction error across laypeople, academics, and practitioners. A between-subjects ANOVA showed a statistically significant difference in prediction error for academics, practitioners, and laypeople (f(2, 1,031) = 168.04, *p* < 0.001, ω^*2*^ = 0.24). Post-hoc comparison *t*-tests, adjusting for a family of three tests using the Holm-Bonferroni correction, revealed statistically significantly higher prediction error among laypeople compared to both academics (*mean difference* = 0.29, *SE* = 0.02, *t*(1,033) = 16.22, *p*_ℎ*olm*_ < 0.001) and practitioners (*mean difference* = 0.25, *SE* = 0.02, *t*(1,033) = 11.30, *p*_ℎ*olm*_ < 0.001). There was no statistically significant difference in prediction error between academics and practitioners (*p*_ℎ*olm*_ = 0.10), though given the smaller samples of the two groups the test may be underpowered to detect a true difference.

In an exploratory follow up analysis examining prediction error by both variable and self-identification, we found the same trend for all of the self-identified groups to show more prediction error for mask-wearing attitudes and intentions compared to signing and sharing the pledge (see Supplemental Information part G).

## Exploratory results

We conducted additional exploratory analyses that were mostly not pre-registered to test whether forecaster individual characteristics such as own political ideology, gender, academic background, and nationality influence the accuracy of forecasters’ predictions. Table 3 presents the results of the individual-level OLS regression of the square prediction error on forecaster characteristics (i.e., regressing prediction error on political ideology, gender, age, academic background, and nationality) and two indicator variables for identifying as a practitioner or an academic on the full sample of forecasters. In the pooled sample of forecasters, having a more conservative (vs. liberal) ideology was statistically significantly associated with higher prediction error or lower accuracy. Being an academic or a practitioner, was also statistically significantly associated with lower prediction error. We also ran exploratory regressions on subsamples of forecasters, specifically laypeople, psychologists, and economists (see Supplemental Information part G).

**Table 3:** Individual level regression of the prediction error on forecaster characteristics.

Exploratory test	Dependent variable:
	Squared prediction error
	Full sample of forecasters
Ideology	0.019* (0.003)
Female	0.016 (0.012)
Age	0.0001 (0.0004)
Background in economics	− 0.022 (0.019)
Background in psychology	− 0.026 (0.020)
American	− 0.004 (0.019)
Practitioner	− 0.081* (0.021)
Academic	− 0.089* (0.020)
Constant	0.074* (0.028)
Observations	1032^a^
R^2^	0.146
Adjusted R^2^	0.139
Residual std. error	0.195 (df = 1023)
F Statistic	21.785* (df = 8; 1023)

*p < 0.05.

^a^Two forecasters didn’t provide information on their political ideology and were dropped from the analysis.

## Discussion

During the COVID-19 pandemic, especially before vaccines had become readily available, it has been considered critical to develop behavioral interventions to nudge citizens to wear masks^36^. The existing vaccination hesitancy paired with the emergence of new coronavirus variants highlights the importance of continued mask-wearing^7,37^. It is thus important to capture the differences in forecasting precision of these interventions for multiple reasons. For example, researchers and policymakers constantly make decisions regarding which intervention to implement. Beliefs that are at odds with the actual success of a nudge could prove costly from a welfare perspective if the wrong intervention is selected. In addition, overestimating the effectiveness of interventions ex ante runs the risk of statistically underpowering them in experimental studies, leading to results that are more likely to be false negative ones in case of null results, and more likely to be false positive, exaggerated or even of the wrong sign compared to the true effect if statistically significant^38^. Alternatively, if forecasters make accurate predictions, predictions of the effectiveness of interventions by political affiliation shines a light on the potential receptiveness to nudges across groups.

More generally, we know little about who can make more accurate forecasts. Do individuals who have a policy-making or academic background make more accurate forecasts for these types of interventions relative to laypeople? In the present forecasting study, both behavioral scientists and laypeople did not anticipate nudges to wear a mask to be ineffective or to backfire. In particular, all forecasters expected nudge interventions to affect Democrats more than Republicans, relative to the control condition. While behavioral scientists were more accurate than laypeople in their forecasts, nevertheless, all of our participant groups were overly optimistic that the nudges would promote mask-wearing, especially among Democrats. Forecasters’ accuracy also varies depending on the outcome that is being predicted, with participants showing more accurate predictions with respect to signing and sharing the pledge compared to predicting attitudes and intentions towards mask-wearing. Interestingly, the forecaster’s own political affiliation had little explanatory power and did not yield substantial variations in estimates for political ingroups or outgroups, which is an interesting finding in the context of the previously discussed political polarization literature^9,11,18,39^.

With the limited foresight indicated by these forecasts—even among professional scientists—there could be large financial and public health costs of betting on ineffective interventions and policies if they are not empirically evaluated. In a polarizing political environment with people’s health and lives at stake, it is important to conduct pre-registered experiments with high statistical power to test the efficacy of public health interventions, thus avoiding ineffective strategies based on inaccurate beliefs and embracing effective ones. One limitation of this study is the extent to which we were able to investigate the ability to predict the success of interventions on actual behavior, as opposed to intentions. Here in our paper, we were limited by the interventions that Gelfand et al.^11^ carried out in their field experiment. Although sharing and signing a pledge contain elements of actual behavior, it is arguably still sufficiently far removed from the targeted behavior of wearing a mask and the existing literature has established that those are related but not completely aligned^2,40^. With that, whether and to what extent the ability to predict interventions that target and measure mask-wearing more directly varies across our target groups of interest remains an empirical question. It is also unclear to what extent forecasters in our study were motivated to perform well when making their forecasts. While we had some monetary incentives, these were small, and only 10 participants (1% of the sample) were randomly chosen for potential payments depending on their performance. Larger monetary incentives might improve forecasting performance, though^23^ find no statistically significant effects on accuracy when forecasters were randomized to monetary incentives or not when making forecasts on conceptual replications. In addition, it is not clear whether prediction markets, where participants bet on the outcome of scientific results and can infer something about the beliefs of other participants based on changes in market prices, would have performed differently. While prediction markets perform relatively well (but far from perfect) for binary replication outcomes (see, e.g.^41^),^26^ find that when it comes to predicting relative effect sizes of replications, the prediction markets performed worse than an unincentivized survey, whereas^42^ find that neither markets nor surveys perform well in predicting new effects in their study (with few participants). Lastly, although we had larger sample sizes than the minimum that we pre-registered, our academic and practitioner samples are smaller than the laypeople sample.

These current limitations can thus be a good starting point for future research. For example, examining the impact of incentives and feedback on accuracy would help us understand whether a forecaster’s ability can be improved in the short term. Alternatively, a long run perspective would be to investigate whether teaching statistical and/or behavioral science knowledge can increase the forecaster’s ability. One could also examine population heterogeneities in forecasting abilities (e.g., repeated exposure to making forecasts) to understand whether the quality of forecasting changes with training, which was not the focus of our investigation. Finally, our work emphasizes the value of academic-practitioner collaborations in the quest of behavior change^37^. Because of our collaboration with various renowned behavioral insights teams that gave us access to their pool of employees in return for consortium co-authorship, we were able to leverage a comprehensive investigation with a diverse set of participants. We believe that this can be a model for future research collaborations.

Collaborations between behavioral science institutions can effectively inform policymaking while accounting for political polarization. In settings where beliefs are largely correct, it could be interesting to explore decision markets. These markets function similarly to prediction markets where participants (e.g., policymakers) bet on the outcomes of the interventions and market prices would help decide which interventions to test and run, where all interventions have a positive probability of being chosen but some get extra weight depending on the specific decision rule by the market designer (e.g. extra weight to interventions that are predicted to have larger effect sizes). Moving forward, we hope that our insights can be useful for the growing field that focuses on predictions of scientific results^21,22^, as well as to behavioral scientists who can use intervention and forecasting data in policymaking to evaluate their current beliefs and inform behavioral policy recommendations^6,43^.

## Supplementary Information

Supplementary Information.
