# Supplementary material for: Politicizing mask-wearing: predicting the success of behavioral interventions among republicans and democrats in the U.S
Source: Sci Rep. 2022 May 9;12:7575. doi: 10.1038/s41598-022-10524-1 (PMC9082983; doi:10.1038/s41598-022-10524-1)
Supplement: Supplementary file 1 — Supplementary Information. [file 41598_2022_10524_MOESM1_ESM.docx]

**Supplemental Information**

for

“Politicizing Mask-Wearing: Predicting the Success of Behavioral Interventions Among Republicans and Democrats”

Dimant et al. (2021)

1. **Original Nudge Conditions (Gelfand et al., 2021)**

**Control (Condition 1)**

This was the baseline condition and included the standard message with no additional justification. The message is as follows:

“Months after the start of the COVID-19 pandemic, many areas of the U.S. are opening to some degree and some segments of the population are starting to move around relatively freely. However, since a cure for COVID-19 has not been found and COVID-19 remains a serious threat, it is important to wear a mask or face covering.”

The image that accompanied the message for this condition was:


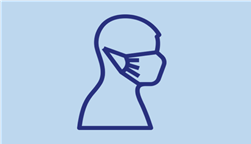


**Protection from Harm (Self) (Condition 2**)

This condition highlighted the liberal moral value ‘harm’ as justification for engaging in prevention behaviors. It specifically stated:

“Months after the start of the COVID-19 pandemic, many areas of the U.S. are opening to some degree and some segments of the population are starting to move around relatively freely. However, since a cure for COVID-19 has not been found and COVID-19 remains a serious threat, it is important to wear a mask or face covering **because it will keep you safe**.”

The image that accompanied the message for this condition was:


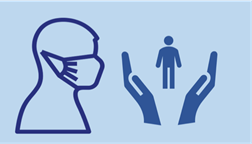


**Protection from Harm (Community) (Condition 3)**

This condition focused on preventing harm to others as the justification for wearing a mask or face covering. This condition included the following message:

“Months after the start of the COVID-19 pandemic, many areas of the U.S. are opening to some degree and some segments of the population are starting to move around relatively freely. However, since a cure for COVID-19 has not been found and COVID-19 remains a serious threat, it is important to wear a mask or face covering **because it will keep our communities safe**.”

The image that accompanied the message for this condition was:

**
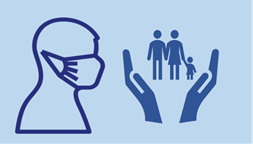
**

**Patriotic Duty (Condition 4)**

This condition was designed to tap into the moral foundation of ‘ingroup-loyalty’ at a broader level—i.e., making patriotic sacrifices for one’s country. This condition included the following message:

“Months after the start of the COVID-19 pandemic, many areas of the U.S. are opening to some degree and some segments of the population are starting to move around relatively freely. However, since a cure for COVID-19 has not been found and COVID-19 remains a serious threat, it is important to wear a mask or face covering **because it is our patriotic duty to make sacrifices for our great country**.”

The image that accompanied the message for this condition was:


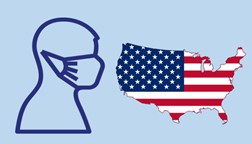


**Purity (Condition 5)**

This condition employed the conservative moral value ‘purity’, which is based in the psychological desire to avoid contamination. This condition included the following message:

“Months after the start of the COVID-19 pandemic, many areas of the U.S. are opening to some degree and some segments of the population are starting to move around relatively freely. However, since a cure for COVID-19 has not been found and COVID-19 remains a serious threat, it is important to wear a mask or face covering **because it will keep our bodies from being contaminated by a disgusting virus**.”

The image that accompanied the message for this condition was:


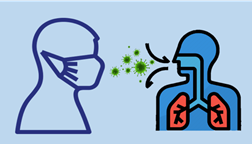


**Reviving the Economy (Condition 6)**

This condition highlighted the importance of following health guidelines for a successful reopening of the economy. Participants read:

“Months after the start of the COVID-19 pandemic, many areas of the U.S. are opening to some degree and some segments of the population are starting to move around relatively freely. However, since a cure for COVID-19 has not been found and COVID-19 remains a serious threat, it is important to wear a mask or face covering **because it will help us to reopen our economy more quickly**.”

The image that accompanied the message for this condition was:


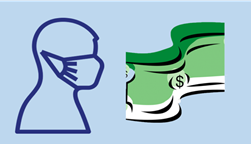


**Threat (Condition 7)**

This condition emphasized the threat that COVID-19 continues to pose to Americans and the severity of the potential consequences of contracting the virus. Participants read:

“Months after the start of the COVID-19 pandemic, many areas of the U.S. are opening to some degree and some segments of the population are starting to move around relatively freely. However, since a cure for COVID-19 has not been found and COVID-19 remains a serious threat, it is important to wear a mask or face covering **because COVID-19 has killed over 211,000 Americans and continues to spread rapidly.**” (Note: we updated the number of deaths at the time of survey launch)

The image that accompanied the message for this condition was:


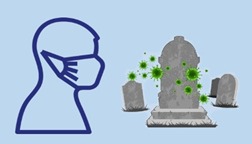


**Scientific Evidence (Condition 8)**

As the messages from health and political officials regarding the importance of masks were unclear and often conflicting during earlier stages of the COVID-19 crisis, this condition emphasized that there is clear scientific evidence showing that masks effectively reduce the spread of the virus.

“Months after the start of the COVID-19 pandemic, many areas of the U.S. are opening to some degree and some segments of the population are starting to move around relatively freely. However, since a cure for COVID-19 has not been found and COVID-19 remains a serious threat, it is important to wear a mask or face covering **because scientific evidence has proven that they can effectively prevent the spread of the virus**.”

The image that accompanied the message for this condition was[MJG1] :


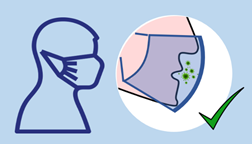


1. **Categorization of Participants**

**Self-Identification Survey Question**

**
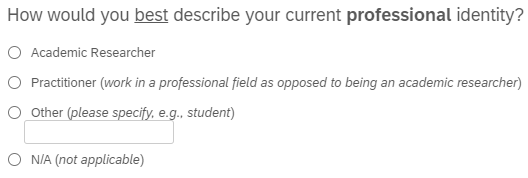
**

**Identification Table**

| **Sample** | **Laypeople** | **Academics** | **Practitioners** |
| --- | --- | --- | --- |
| **Qualtrics** | All participants irrespective of their self-identification or education. | None | None |
| **Professional Networks**  **(Social Media; Behavioral Science Units; Social Science Prediction Platform)** | Participants who self-identified as a layperson (“other” or “N/A” responses). | Participants who self-identified as an “Academic Researcher” with a four-year college degree or higher. | Participants who self-identified as a “Practitioner” with a four-year college degree or higher. |

1. **Sample Characteristics**

**Sample Demographics: Academics vs. Pracitioners**

| Name | Academics  (*n* = 199) | Pracitioners  (*n* = 121) |
| --- | --- | --- |
| Age in Years | 35.35 (*10.32*) | 35.80 (*10.12*) |
| Family Income* | 3.58 (*1.58*) | 3.92 (*1.48*) |
| Socioeconomic Status (SES) | 8.60 (*1.49*) | 8.50 (*1.51*) |
| Ideology (Liberal to Conservative) | 3.06 (*1.38*) | 3.16 (*1.49*) |
| Gender |  |  |
| Woman | 121 (61%) | 62 (51%) |
| Man | 76 (38%) | 58 (48%) |
| Other | 1 (1%) | 1 (1%) |
| Race |  |  |
| White | 160 (80%) | 92 (75%) |
| Black | 1 (1%) | 2 (2%) |
| Hispanic | 11 (6%) | 5 (4%) |
| Asian | 17 (9%) | 15 (12%) |
| Multiracial | 2 (1%) | 5 (4%) |
| Pacific Islander | - | 1 (1%) |
| Other | 8 (4%) | 1 (1%) |
| Highest Education |  |  |
| Graduate (4-year) | 18 (9%) | 38 (31%) |
| Professional Degree (Ph.D., M.A., etc.) | 181 (91%) | 83 (69%) |
| Area |  |  |
| Urban | 119 (60%) | 84 (69%) |
| Suburban | 64 (32%) | 34 (28%) |
| Rural | 16 (8%) | 3 (3%) |
| Field |  |  |
| Psychology | 48 (24%) | 20 (17%) |
| Economics | 85 (43%) | 23 (19%) |
| Multiple Degrees | 26 (13%) | 18 (15%) |
| Other | 40 (20%)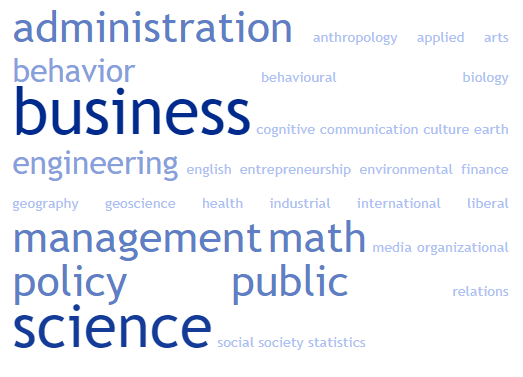 | 59 (49%)  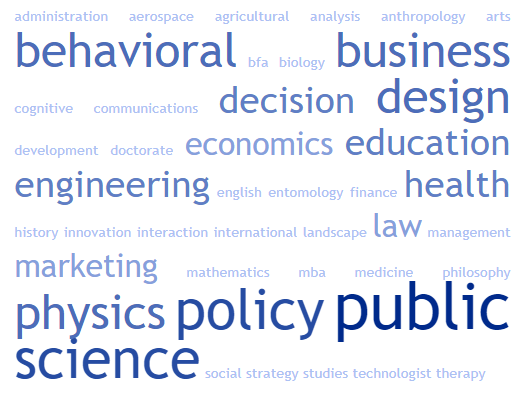 |

*Note*: Values are means (*SDs*) or counts (frequencies) unless otherwise noted.

* 1 = “Below 30k”, 2 = “30-60k”, 3 = “60-90k”, 4 = “90-120k”, 5 = “Above 120k”.

1. **Format of Forecasted Predictions**


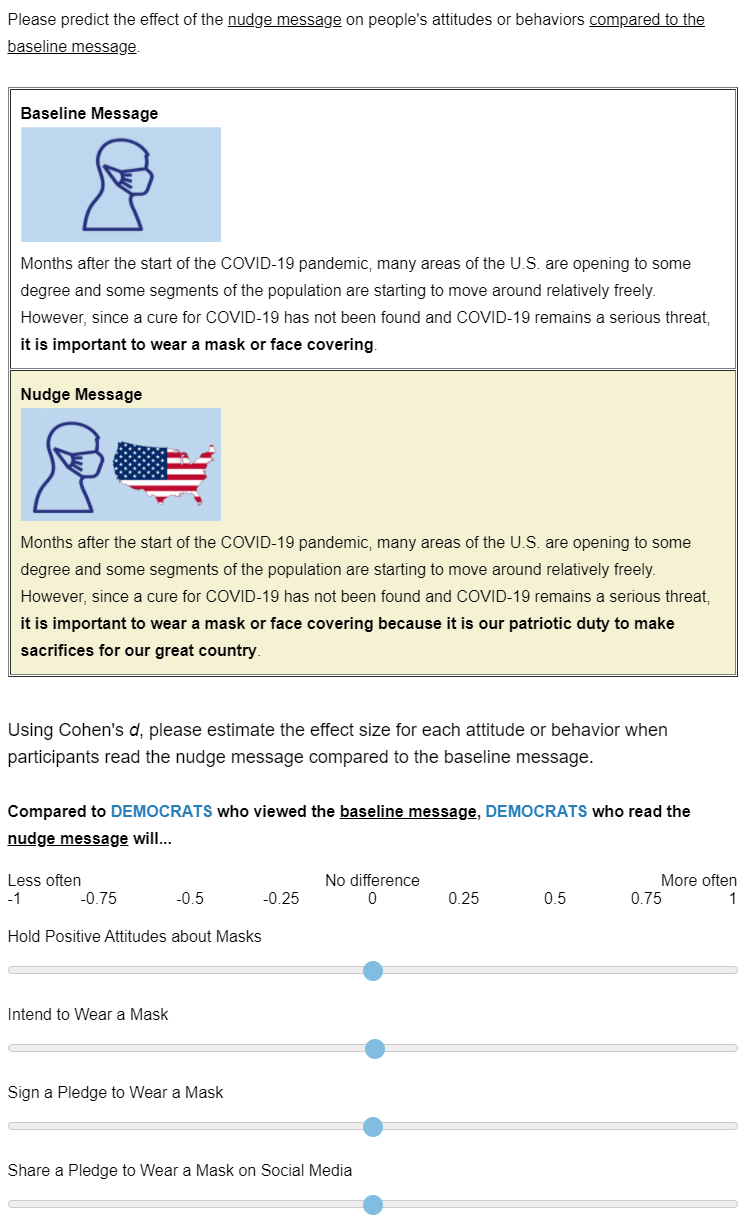


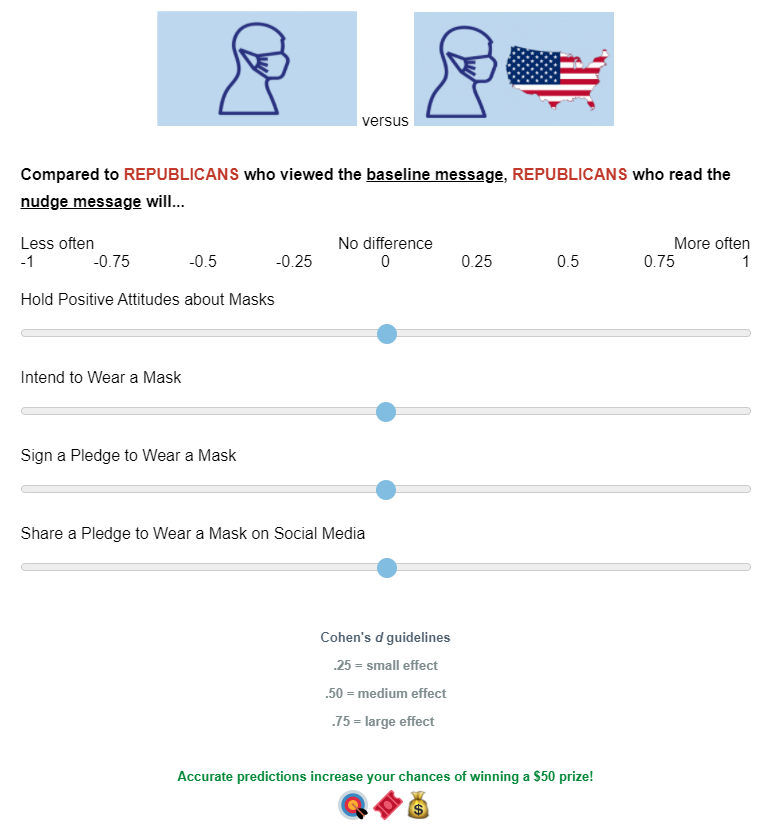


1. **Deviations from the Pre-Registration**
2. In the pre-registration plan for hypothesis 4, we mistakenly specified that we would estimate the mean predicted nudge effect for the 56 nudge predictions made by that for each forecaster, and then compare the mean of this individual level variable (*N* = number of forecasters in the data = 1,031) to the mean observed nudge effects (*N* = number of nudge effects = 7) in a *z*-test. However, such a test is in fact impossible. We therefore estimated the mean predicted nudge effect for each nudge and forecaster (*N* = 7 * number of forecasters = 7,217) and compared these 7 individual level means to the observed nudge effects (*N* = number of nudges = 7) in a *z*-test.
3. In the pre-registration plan for hypothesis 6, we mistakenly specified that we would estimate the mean predicted nudge effect across laypeople, academics, and practitioners using a repeated-measures ANOVA. However, since hypothesis 6 was written as between-subjects and did not specify tests of repeated measures, we estimated the mean predicted nudge effect across laypeople, academics, and practitioners using a between-subjects ANOVA.
4. As an exploratory test, we pre-registered that we would regress the squared prediction errors for the seven nudges for each forecaster on demographic variables and individual fixed effects. Given that demographic variables are constant across nudges, they are dropped when including individual fixed effects. We hence instead regress the mean squared prediction error for each forecaster on individual-level demographic characteristics.
5. **Statistics for the Calculation of the Observed Effect Sizes**

The first 3 tables show the dependent variables separately, while the last table pools the dependent variables. Attitudes and intentions were measured on 7-point scale (positive values indicate positive attitudes and intentions to wear a mask). Signing and sharing the pledge to wear a mask were measured as binary variables (0 = *did not sign/share*, 1 = *signed/shared*). Effect sizes were calculated using <https://doomlab.shinyapps.io/mote/> (“Mean Differences” 🡪 “Independent t – Means”).

**Descriptive Statistics on Outcomes across Nudges (Pooled Sample)**

| **Nudge x Outcome** | ***n*** | **Mean** | ***SD*** | ***SE*** |
| --- | --- | --- | --- | --- |
| Control | 614 |  |  |  |
| Attitude |  | 6.18 | 1.43 | 0.06 |
| Intention |  | 6.18 | 1.34 | 0.05 |
| Signed Pledge |  | 0.64 | 0.48 | 0.02 |
| Shared Pledge |  | 0.21 | 0.41 | 0.02 |
| USA | 614 |  |  |  |
| Attitude |  | 6.08 | 1.50 | 0.06 |
| Intention |  | 6.18 | 1.31 | 0.05 |
| Signed Pledge |  | 0.62 | 0.49 | 0.02 |
| Shared Pledge |  | 0.21 | 0.41 | 0.02 |
| Purity | 620 |  |  |  |
| Attitude |  | 6.15 | 1.49 | 0.06 |
| Intention |  | 6.19 | 1.36 | 0.05 |
| Signed Pledge |  | 0.62 | 0.49 | 0.02 |
| Shared Pledge |  | 0.20 | 0.40 | 0.02 |
| Economy | 613 |  |  |  |
| Attitude |  | 6.20 | 1.41 | 0.06 |
| Intention |  | 6.24 | 1.28 | 0.05 |
| Signed Pledge |  | 0.64 | 0.48 | 0.02 |
| Shared Pledge |  | 0.21 | 0.41 | 0.02 |
| Harm (Self) | 620 |  |  |  |
| Attitude |  | 6.16 | 1.45 | 0.06 |
| Intention |  | 6.22 | 1.32 | 0.05 |
| Signed Pledge |  | 0.66 | 0.47 | 0.02 |
| Shared Pledge |  | 0.22 | 0.42 | 0.02 |
| Harm (Community) | 613 |  |  |  |
| Attitude |  | 6.20 | 1.39 | 0.06 |
| Intention |  | 6.16 | 1.39 | 0.06 |
| Signed Pledge |  | 0.65 | 0.48 | 0.02 |
| Shared Pledge |  | 0.21 | 0.41 | 0.02 |
| Scientific Evidence | 619 |  |  |  |
| Attitude |  | 6.05 | 1.58 | 0.06 |
| Intention |  | 6.06 | 1.49 | 0.06 |
| Signed Pledge |  | 0.60 | 0.49 | 0.02 |
| Shared Pledge |  | 0.19 | 0.39 | 0.02 |
| Threat | 618 |  |  |  |
| Attitude |  | 6.17 | 1.39 | 0.06 |
| Intention |  | 6.23 | 1.26 | 0.05 |
| Signed Pledge |  | 0.58 | 0.49 | 0.02 |
| Shared Pledge |  | 0.23 | 0.42 | 0.02 |

**Descriptive Statistics on Outcomes across Nudges (Democrats)**

| **Nudge x Outcome** | ***n*** | **Mean** | ***SD*** | ***SE*** |
| --- | --- | --- | --- | --- |
| Control | 310 |  |  |  |
| Attitude |  | 6.70 | 0.62 | 0.04 |
| Intention |  | 6.65 | 0.67 | 0.04 |
| Signed Pledge |  | 0.71 | 0.46 | 0.03 |
| Shared Pledge |  | 0.24 | 0.42 | 0.02 |
| USA | 307 |  |  |  |
| Attitude |  | 6.68 | 0.62 | 0.04 |
| Intention |  | 6.67 | 0.60 | 0.03 |
| Signed Pledge |  | 0.72 | 0.45 | 0.03 |
| Shared Pledge |  | 0.27 | 0.45 | 0.03 |
| Purity | 311 |  |  |  |
| Attitude |  | 6.64 | 0.77 | 0.04 |
| Intention |  | 6.63 | 0.69 | 0.04 |
| Signed Pledge |  | 0.72 | 0.45 | 0.03 |
| Shared Pledge |  | 0.25 | 0.43 | 0.02 |
| Economy | 305 |  |  |  |
| Attitude |  | 6.66 | 0.74 | 0.04 |
| Intention |  | 6.65 | 0.66 | 0.04 |
| Signed Pledge |  | 0.73 | 0.45 | 0.03 |
| Shared Pledge |  | 0.27 | 0.44 | 0.03 |
| Harm (Self) | 310 |  |  |  |
| Attitude |  | 6.68 | 0.71 | 0.04 |
| Intention |  | 6.67 | 0.69 | 0.04 |
| Signed Pledge |  | 0.76 | 0.42 | 0.02 |
| Shared Pledge |  | 0.26 | 0.44 | 0.03 |
| Harm (Community) | 306 |  |  |  |
| Attitude |  | 6.67 | 0.60 | 0.03 |
| Intention |  | 6.58 | 0.76 | 0.04 |
| Signed Pledge |  | 0.71 | 0.45 | 0.03 |
| Shared Pledge |  | 0.24 | 0.43 | 0.02 |
| Scientific Evidence | 312 |  |  |  |
| Attitude |  | 6.60 | 0.84 | 0.05 |
| Intention |  | 6.56 | 0.84 | 0.05 |
| Signed Pledge |  | 0.69 | 0.46 | 0.03 |
| Shared Pledge |  | 0.21 | 0.40 | 0.02 |
| Threat | 307 |  |  |  |
| Attitude |  | 6.62 | 0.75 | 0.04 |
| Intention |  | 6.62 | 0.68 | 0.04 |
| Signed Pledge |  | 0.65 | 0.48 | 0.03 |
| Shared Pledge |  | 0.29 | 0.45 | 0.03 |

**Descriptive Statistics on Outcomes across Nudges (Republicans)**

| **Nudge x Outcome** | ***n*** | **Mean** | ***SD*** |  | ***SE*** |
| --- | --- | --- | --- | --- | --- |
| Control | 304 |  |  |  |  |
| Attitude |  | 5.65 | 1.78 |  | 0.10 |
| Intention |  | 5.71 | 1.64 |  | 0.09 |
| Signed Pledge |  | 0.57 | 0.50 |  | 0.03 |
| Shared Pledge |  | 0.18 | 0.39 |  | 0.02 |
| USA | 307 |  |  |  |  |
| Attitude |  | 5.49 | 1.85 |  | 0.11 |
| Intention |  | 5.68 | 1.60 |  | 0.09 |
| Signed Pledge |  | 0.51 | 0.50 |  | 0.03 |
| Shared Pledge |  | 0.15 | 0.35 |  | 0.02 |
| Purity | 309 |  |  |  |  |
| Attitude |  | 5.66 | 1.85 |  | 0.11 |
| Intention |  | 5.74 | 1.69 |  | 0.10 |
| Signed Pledge |  | 0.53 | 0.50 |  | 0.03 |
| Shared Pledge |  | 0.15 | 0.36 |  | 0.02 |
| Economy | 308 |  |  |  |  |
| Attitude |  | 5.73 | 1.73 |  | 0.10 |
| Intention |  | 5.84 | 1.58 |  | 0.09 |
| Signed Pledge |  | 0.56 | 0.50 |  | 0.03 |
| Shared Pledge |  | 0.16 | 0.36 |  | 0.02 |
| Harm (Self) | 310 |  |  |  |  |
| Attitude |  | 5.63 | 1.78 |  | 0.10 |
| Intention |  | 5.77 | 1.61 |  | 0.09 |
| Signed Pledge |  | 0.55 | 0.50 |  | 0.03 |
| Shared Pledge |  | 0.18 | 0.38 |  | 0.02 |
| Harm (Community) | 307 |  |  |  |  |
| Attitude |  | 5.73 | 1.75 |  | 0.10 |
| Intention |  | 5.74 | 1.71 |  | 0.10 |
| Signed Pledge |  | 0.59 | 0.49 |  | 0.03 |
| Shared Pledge |  | 0.19 | 0.39 |  | 0.02 |
| Scientific Evidence | 307 |  |  |  |  |
| Attitude |  | 5.49 | 1.92 |  | 0.11 |
| Intention |  | 5.55 | 1.81 |  | 0.10 |
| Signed Pledge |  | 0.50 | 0.50 |  | 0.03 |
| Shared Pledge |  | 0.17 | 0.38 |  | 0.02 |
| Threat | 311 |  |  |  |  |
| Attitude |  | 5.72 | 1.69 |  | 0.10 |
| Intention |  | 5.85 | 1.55 |  | 0.09 |
| Signed Pledge |  | 0.51 | 0.50 |  | 0.03 |
| Shared Pledge |  | 0.39 | 0.39 |  | 0.02 |

**Descriptive Statistics on Pooled Prediction Error across Nudges and Samples**

| **Nudge x Sample** | ***n*** | **Mean** | ***SD*** | ***SE*** |
| --- | --- | --- | --- | --- |
| Pooled Sample |  |  |  |  |
| Control | 614 | 0.01 | 0.71 | 0.03 |
| USA | 614 | -0.02 | 0.73 | 0.03 |
| Purity | 620 | -0.01 | 0.72 | 0.03 |
| Economy | 613 | 0.03 | 0.67 | 0.03 |
| Harm (Self) | 620 | 0.03 | 0.71 | 0.03 |
| Harm (Community) | 613 | 0.02 | 0.69 | 0.03 |
| Scientific Evidence | 619 | -0.07 | 0.74 | 0.03 |
| Threat | 618 | 0 | 0.68 | 0.03 |
| Democrats |  |  |  |  |
| Control | 310 | 0.24 | 0.44 | 0.03 |
| USA | 307 | 0.27 | 0.47 | 0.03 |
| Purity | 311 | 0.24 | 0.47 | 0.03 |
| Economy | 305 | 0.26 | 0.45 | 0.03 |
| Harm (Self) | 310 | 0.29 | 0.46 | 0.03 |
| Harm (Community) | 306 | 0.22 | 0.46 | 0.03 |
| Scientific Evidence | 312 | 0.18 | 0.49 | 0.03 |
| Threat | 307 | 0.22 | 0.50 | 0.03 |
| Republicans |  |  |  |  |
| Control | 304 | -0.22 | 0.84 | 0.05 |
| USA | 307 | -0.30 | 0.82 | 0.05 |
| Purity | 309 | -0.25 | 0.83 | 0.05 |
| Economy | 308 | -0.21 | 0.77 | 0.04 |
| Harm (Self) | 310 | -0.23 | 0.81 | 0.05 |
| Harm (Community) | 307 | -0.19 | 0.81 | 0.05 |
| Scientific Evidence | 307 | -0.32 | 0.86 | 0.05 |
| Threat | 311 | -0.21 | 0.76 | 0.04 |
| *Note*. Outcomes were centered and scaled, then averaged. | | | | |

1. **Prediction Error by Self-Identification and Variable**

**
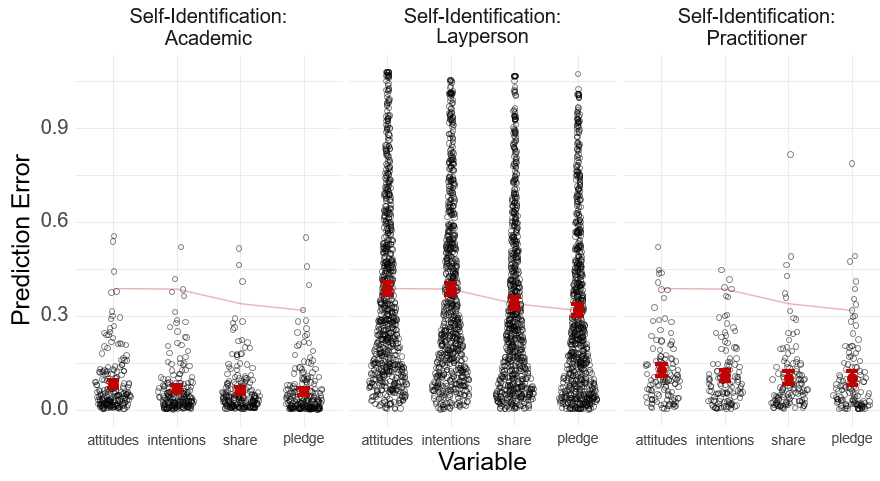
**

1. **Exploratory Analyses by Subsample**

Columns (1), (2) and (3) estimate the regression separately for laypeople and academics and practitioners with a background in psychology or economics (not pre-registered). In the sample of laypeople, having a more conservative ideology was statistically significantly associated with higher prediction error, while a higher level of education was statistically significantly associated with lower prediction error. Being a woman and having higher income was not statistically significantly associated with accuracy. Being a woman was statistically significantly associated with higher prediction error or lower accuracy in both samples of forecasters with a background in psychology or economics, while being American was associated with lower prediction error only among economists. In addition, having a more conservative ideology was associated with higher prediction error or lower accuracy in the economics samples. Academics showed statistically significantly lower prediction error or higher accuracy than practitioners only in the economics subsample.

| **Additional exploratory tests** | | | |
| --- | --- | --- | --- |
|  | | | |
|  | *Dependent variable:* | | |
|  |  | | |
|  | Squared prediction error | | |
|  | Laypeople | Psychology only | Economics only |
|  | (1) | (2) | (3) |
|  | | | |
| Ideology | 0.019^*^ | 0.002 | 0.007^*^ |
|  | (0.003) | (0.003) | (0.003) |
|  |  |  |  |
| Female | 0.010 | 0.034^*^ | 0.022^*^ |
|  | (0.018) | (0.009) | (0.007) |
|  |  |  |  |
| Age | 0.0004 | -0.0002 | -0.001 |
|  | (0.0005) | (0.0004) | (0.0004) |
|  |  |  |  |
| Income | 0.0004 |  |  |
|  | (0.006) |  |  |
|  |  |  |  |
| Education level | -0.010^*^ |  |  |
|  | (0.005) |  |  |
|  |  |  |  |
| American |  | -0.017 | -0.023^*^ |
|  |  | (0.010) | (0.007) |
|  |  |  |  |
| Academic |  | -0.008 | -0.019^*^ |
|  |  | (0.010) | (0.008) |
|  |  |  |  |
| Constant | 0.100^*^ | 0.037 | 0.045^*^ |
|  | (0.040) | (0.020) | (0.017) |
|  |  |  |  |
|  | | | |
| Observations | 713 | 97 | 133 |
| R^2^ | 0.061 | 0.189 | 0.208 |
| Adjusted R^2^ | 0.054 | 0.144 | 0.176 |
| Residual Std. Error | 0.230 (df = 707) | 0.044 (df = 91) | 0.040 (df = 127) |
| F Statistic | 9.152^*^ (df = 5; 707) | 4.241^*^ (df = 5; 91) | 6.657^*^ (df = 5; 127) |
|  | | | |
| *Note:* *p<0.05. | | | |
